# lncRNA STAT4-AS1 Inhibited TH17 Cell Differentiation by Targeting ROR*γ*t Protein

**DOI:** 10.1155/2022/8307280

**Published:** 2022-04-28

**Authors:** Hanlin He, Xiangjie Qiu, Mingming Qi, Ousman Bajinka, Ling Qin, Yurong Tan

**Affiliations:** ^1^Department of Medical Microbiology, Central South University, Changsha, 410078 Hunan, China; ^2^Department of Obstetrics, Zhuzhou Hospital Affiliated to Xiangya School of Medicine, Central South University, Hunan, China; ^3^China-Africa Research Center of Infectious Diseases, Central South University, Changsha, 410078 Hunan, China; ^4^Department of Respiratory Medicine, National Key Clinical Specialty, Branch of National Clinical Research Center for Respiratory Disease, National Clinical Research Center for Geriatric Disorders; Hunan Provincial Clinical Research Center for Respiratory Diseases, Xiangya Hospital, Central South University, Changsha, Hunan, China

## Abstract

**Objective:**

From our previous study, we obtained long noncoding RNA (lncRNA) STAT4-AS1, which is related to asthma through high-throughput screening. However, we could not determine the specific mechanism involved and in response to this. We further designed this study.

**Results:**

First, we found that lncRNA STAT4-AS1 was downregulated in T cells from patients with asthma when compared to healthy controls. Next, we confirmed that lncRNA STAT4-AS1 was significantly negatively correlated with T helper 17 (TH17) differentiation in vitro experiments. The decreases of STAT4-AS1 promoted TH17 differentiation, while the increases of STAT4-AS1 inhibited TH17 differentiation. Subsequently, through RNA pull-down, RNA-binding protein immunoprecipitation (RIP), and dual luciferase reporter assay, we found that STAT4-AS1 could inhibit the binding of retinoid-related orphan receptor-*γ*t (ROR*γ*t) protein with an IL-17A promoter after binding with ROR*γ*t protein. Fluorescence in situ hybridization (FISH) and nuclear-cytoplasmic separation assay showed that STAT4-AS1 is bonded to ROR*γ*t in the cytoplasm, preventing ROR*γ*t from entering the nucleus.

**Conclusion:**

Overall, STAT4-AS1 directly targets ROR*γ*t protein, inhibits the mutual binding of ROR*γ*t and IL-17 gene promoter, and eventually inhibits TH17 differentiation. To this end, STAT4-AS1 as a potential target may confer applications in the clinical treatment and diagnosis of TH17-related diseases.

## 1. Introduction

The differentiation disorder of TH17 cell is recent been studied with growing evidence as to its association with many autoimmune diseases such as multiple sclerosis (MS), rheumatoid arthritis (RA), psoriasis, Crohn's disease (CD), obesity, and metabolic syndromes [1, 31, 42]. Asthma can be mediated by a variety of mechanisms including airway immune imbalance and pulmonary brain axis [2, 8]. However, an increased IL-17A signal, which activates neutrophils and macrophages, is the main mechanism of refractory asthma [30]. Moreover, worsening of acute asthma, exacerbation of symptoms of acute asthma, and the airways of patients with severe persistent asthma also have increased IL-17A signals. Nocturnal asthma attacks are also associated with enhanced TH17. After the stimulation of T cell receptors, the initial CD4^+^ T cells are induced to differentiate into TH17 cells by TGF-*β*, IL-6, IL-23, IL-21, IL-1*β*, and STAT3, producing the signature cytokines IL-17A, IL-17F, IL-21, and IL-22 [37]. These cytokines activate many types of cells such as epithelial cells, endothelial cells, and fibroblasts to secrete IL-6, IL-8, G-CSF, ICAM-1, and the likes, and they promote inflammatory response through MAP kinase pathway and NF-*κ*B pathway [7, 16]. Moreover, IL-17A can mobilize, recruit, and activate inflammatory cells, especially neutrophils to release inflammatory cytokines, thereby inducing the secretion of mucus and ultimately airway remodeling [3, 20, 24]. In a mouse model of asthma, the deficiency of IL-17A receptor reduced the recruitment of antigen-induced neutrophils and eosinophils and also reduced the airway inflammatory response. Other studies also reported that the airway hyperresponsiveness (AHR) was significantly inhibited in the IL-17A knockout mouse model [17, 19].

The mechanism underpinning of TH17 cell development and differentiation is extremely complex. ROR*γ*t is the major transcription factor in humans and mice that do not only promote the differentiation of TH17 cells but also regulates the expression and secretion of IL-17A [27, 28]. ROR*γ*t (ROR*γ*2) and its isoform ROR*γ* (ROR*γ*) are encoded by a single gene called Rorg (also known as Rorc). ROR*γ*t is a member of the orphan nuclear receptor family of retinoic acid, expressed on TH17 cells and thymocytes, and found ubiquitously in mice and humans. ROR*γ*t binds to the ROR response elements (Rore) within the Il17a promoter in mice and humans [18]. Luciferase reporter assays and chromatin conformation capture analysis have shown that ROR*γ*t binds to Rore and mediates IL-17A transcription by controlling chromatin remodeling [41]. Chromatin immunoprecipitation (ChIP) analysis showed that the Runt-related transcription factor (RUNX) 1 formed a complex with ROR*γ*t, and bound the IL-17A promoter [45]. T cells without ROR*γ*t were unable to differentiate into TH17 *in vitro* [10]. It has been found that digoxin could effectively delay and reduce the severity of autoimmune diseases in mice such as MS and experimental autoimmune encephalomyelitis. It does this through changing the active conformation of ROR*γ*t and inhibiting the differentiation of TH17 cells [13]. This study suggested that targeting ROR*γ*t to inhibit the differentiation and function of TH17 cells is a promising treatment for TH17-mediated inflammation or autoimmune diseases.

lncRNAs are noncoding RNAs whose transcriptional lengths are more than 200 nucleotides. They confer little or may not be involved in protein coding due to the lack of an open reading framework. In our previous work, we obtained lncRNA STAT4-AS1 related to asthma through high-throughput screening. lncRNA STAT4-AS1 is a freshly categorized lncRNA that is decreasingly expressed in asthma. However, its biological functions and fundamental molecular mechanisms are completely undefined. Herein, we designed to confirm the regulatory role of lncRNA STAT4-AS1 in the differentiation of TH17 and provide scientific basis for new therapeutic targets for TH17-related diseases.

## 2. Materials and Methods

### 2.1. Bioinformatics Analysis

The sequence of lncRNA STAT4-AS1 was obtained from UCSC Genome Browser (http://genome.ucsc.edu/) and submitted to the CatRapid public database (http://s.tartaglialab.com/page/catrapid_group) and MEM Database (https://biit.cs.ut.ee/mem/) to predict its interacting proteins.

### 2.2. Patients and Health Control

A total of 10 patients (aged 56.1 ± 5.5 years) with refractory asthma were recruited from Xiangya Hospital of Central South University. All patients with refractory asthma were diagnosed by physicians according to the revised version of Chinese guidelines for the prevention and management of bronchial asthma. In addition, 6 healthy volunteers (aged 55.8 ± 3.2) without any immune-related disease or respiratory illness were recruited as normal controls.

The study protocols were approved by the ethical committee of the Xiangya Hospital of Central South University. All participants provided written informed consent. The clinical information of all participants is shown in Supplemental Table 1.

### 2.3. Isolation and Culture of Peripheral Blood Mononuclear Cells (PBMCs)

The peripheral blood samples collected by the subjects were preserved in tubes with pretreatment of heparin sodium. PBMCs were isolated in Ficoll-Hypaque solution by density gradient centrifugation. The isolated PBMCs were routinely cultured in Roswell Park Memorial Institute-1640 (RPMI-1640) culture medium (HyClone, Logan, USA) containing 20% fetal bovine serum and 1% penicillin-streptomycin at 37°C and 5% CO_2_.

### 2.4. TH17 Cell Differentiation

PBMCs (2 × 10^6^ cells/mL) were cultured in 24-well plates containing plate bound anti-CD3 (5 *μ*g/mL) and soluble anti-CD28 (2 *μ*g/mL). Cultures were supplemented with 20 ng/mL of IL-6, 5 ng/mL of TGF-*β*, 25 ng/mL of IL-23, 5 *μ*g/mL of anti-IFN-*γ*, and 5 *μ*g/mL of anti-IL-4 for 4 days. CD3, CD28, TGF-*β*, and IL-23 were purchased from Sino Biological in Wuhan, China, while anti-IFN-*γ* and anti-IL-4 were purchased from Bioxcell in West Lebanon, USA.

### 2.5. Enzyme-Linked Immunosorbent Assay (ELISA)

IL-17 is the most common specific cytokine secreted by TH17 cells, and the TH17/IL-17A axis plays an important role in the pathogenesis of asthma. Thus, IL-17A were selected to detect the effect of lncRNA-STAT4-AS1 in TH17 cell differentiation [5]. The concentrations of IL-17A in the supernatant of PBMC cultured *in vitro* were measured by using ELISA according to the manufacturer's instructions (Fankew, Shanghai, China). In short, the supernatant was added to a 96-well plate with 100 *μ*L per well. Appropriate biotin-binding antibodies (Fankew, Shanghai, China) were added to each well and incubated at room temperature for 2 hours. After washing five times, substrate solution was added to each well and incubated in darkness at room temperature for 30 minutes. The optical density (OD) of each well was detected at a wavelength of 450 nm. The concentration of IL-17A in the sample was calculated according to the standard curve. The specificity of the kit is 99%. All the measurement data were distributed in the standard curve. The kit provided the negative quality control and the positive standard for preparing the standard curve. All ELISA results were expressed as cytokine concentrations (ng/L) and performed in triplicates.

### 2.6. Real-Time Quantitative PCR (RT-qPCR)

Total RNA in the cells was extracted with TRIzol reagent (TaKaRa, Beijing, China) according to the manufacturer's instructions. The concentration of the extracted RNA was detected by a spectrophotometer. RNA was reverse transcribed into cDNA using reverse transcription kits (TaKaRa, Beijing, China) according to the procedure of product specification. The obtained cDNA was detected by RT-qPCR using SYBR®-Green and fluorescent quantitative PCR detection system (Bimake, Houston, USA) according to the procedure of product specification. GAPDH was often used as an internal reference gene in human T cell studies [22, 23, 32]. GAPDH mRNA expression remained stable in this study. Therefore, the mRNA expression levels of candidate genes were normalized to GAPDH. We assumed that the efficiency of RT-qPCR assays was 100%. The relative mRNA levels of candidate genes were analyzed by the 2−*ΔΔ*Ct method [25]. All the primers were synthesized by Sangon Biotech in Shanghai, and the primer sequences used are listed in Supplemental Table 2.

### 2.7. Cell Transfection

To upregulate lncRNA STAT4-AS1 level, lentivirus vectors containing pcDNA-STAT3-AS1 (GenePharma, Shanghai, China) were transfected into PBMCs. Two mL of PBMCs suspension was inoculated into a six-well culture plate for 24 hours before transfection. Ten *μ*L lentiviruses was added to each well, and then 1 *μ*L of polybrene (GenePharma, Shanghai, China) was added to improve transfection efficiency for 24 hours. Empty lentivirus vectors were used as negative controls. To downregulate lncRNA STAT4-AS1 level, 10 *μ*L of lentivirus vectors containing shRNA-STAT4-AS1 (Jtsbio, Wuhan, China) and 1 *μ*L of polybrene (Jtsbio, Wuhan, China) were added to each well for 24 hours. The sequence of the ShRNA-STAT4-AS1 was 5′-GAACATACTGCAGTTTCAA-3′, and the sequence of the ShRNA-NC was 5′-TTCTCCGAACGTGTCACGT-3′.

### 2.8. Flow Cytometry

The differentiated T cells were incubated for 4 hours at 37°C in 5% CO_2_ with 0.1 mg/mL of monensin (Sigma, Saint Louis, USA), 1 *μ*g/mL of ionomycin (Sigma, Saint Louis, USA), and 500 ng/mL of sparfloxacin (Sigma, Saint Louis, USA). Then, cells were stained with FITC-IL-17A monoclonal antibody (BD Bioscience, Franklin, USA) and APC-Cy™ 7-CD4 monoclonal antibody (BD Bioscience, Franklin, USA). The flow cytometry was performed in Cytek Athena system (Cytek, San Francisco, USA). FlowJo software was used to analyze the results.

### 2.9. Immunofluorescence

The induced T cells were fixed in 4% paraformaldehyde (PFA) for 10 minutes, washed with PBS, permeated with PBS+0.2% Triton X-100 (PBTX) for 10 minutes, and then blocked in 10% goat serum for 1 hour. The cells were incubated overnight at 4°C with rabbit anti-ROR*γ*t (GeneTex, Irvine, USA). The following day, the cells were washed with phosphate-buffered saline (PBS) containing 0.3% Triton X-100, blocked in 10% goat serum (GS) for 1 hour and then counterstained with DAPI for 10 minutes. The images were collected under a fluorescence microscope after the slides were sealed with a fluorescence quenching agent.

### 2.10. RNA Pull-Down Experiment

To verify the interaction between ROR*γ*t and lncRNA STAT4-AS1, the RNA pull-down was carried out. lncRNA STAT4-AS1 was synthesized *in vitro* (Genscript, Nanjing, China) and biotin-labeled using Pierce™ RNA3′ End Desthiobiotinylation Kit. Cell extracts were incubated with biotin-labeled lncRNA STAT4-AS1 at 4°C for 1 h. Then, streptavidin magnetic beads (New England Biolabs, USA) were added and incubated at room temperature for 1 h. Lysate proteins in each reaction were detected by Western blot using mouse-anti human ROR*γ*t primary antibody (BD sciences, SD, USA) and goat-anti mouse secondary antibody (Boster, Wuhan, China).

### 2.11. RNA-Binding Protein Immunoprecipitation (RIP) Assay

The RNA immunoprecipitation (RIP) assay was performed using a Magna RIP™ RNA-Binding Protein Immunoprecipitation Kit (Millipore, Billerica, USA). In brief, the PBMCs (1 × 10^7^) in 24-well plates were lysed with an RIP lysis buffer. A part of the cell lysate was used as the input. The remaining cell lysate was divided into two parts and incubated with anti-ROR*γ*t (GeneTex, Irvine, USA), Ago2 (Boster, Wuhan, China), and anti-IgG (Boster, Wuhan, China) at 4°C overnight. Then, the RNA-protein complex was extracted with magnetic beads and proteinase K was used to digest the protein and purify the RNAs. The enrichment of lncRNA STAT4-AS1 in the RNA samples was detected by RT-qPCR. Anti-IgG was used as the negative control for the target antibodies.

### 2.12. Dual Luciferase Reporter Assay

The IL-17 promoter was cloned into the luciferase expression reporter plasmid to construct pGL3-IL-17 (Genscript, Nanjing, China). lncRNA STAT4-AS1 and ROR*γ*t were cloned into pcDNA3.1 to construct pcDNA3.1-RORyt and pcDNA3.1-ROR*γ*t (Genscript, Nanjing, China). After 293 cells were cultured in a 12-well plate for 24 hours (80% confluence), pGL3-IL-17, ROR*γ*t, and lncRNA STAT4-AS1 were cotransfected into 293T cells in different combinations. The fluorescence signals were detected 48 h after transfection according to the manufacturer's instruction, and relative fluorescence values (luciferase/renilla) were calculated.

### 2.13. Fluorescence In Situ Hybridization (FISH)

RNA-FISH was performed with lncRNA STAT4-AS1-specific probes (Sangon Biotech, Shanghai, China), and the probe sequence was designed and synthesized as 5′-CAGCCACCTGACTTCATGCCCTCTTGTTATCTC-3′. The probe signals were detected with a FISH Kit (Boster, Wuhan, China) according to the manufacturer's instruction. Briefly, the cells were fixed with 4% paraformaldehyde for 15 min and then permeated with 0.4% Triton X-100 for 15 min. After prehybridization, the cells were hybridized at 37°C overnight with lncRNA STAT4-AS1 probes. Then, the cells were stained with 4′,6-diamino-2-phenylindole (DAPI) and imaged with an Olympus FV1000 confocal microscope. This assay was repeated three times.

### 2.14. Nuclear-Cytoplasmic Separation Assay

The nuclear-cytoplasmic separation assay was prepared using a nuclear and cytoplasmic Extraction Reagent kit (Bestbio, Shanghai, China) according to the manufacturer's instruction. Briefly, the treated cells were washed twice with cold PBS and centrifuged at 500 g for 3 min. Then, the cell pellet was suspended in 400 *μ*L of extraction reagent A and incubated on ice for 30 min. After centrifuged at 500 g for 5 min, the supernatant fraction containing the cytoplasmic component was transferred to a new tube. The insoluble nuclear fraction was wash once with PBS and suspended in 200 *μ*L of reagent B. Both extractions were used for RNA extraction immediately.

### 2.15. Statistical Analysis

All experiments were independently repeated at least three times. The data was expressed as the mean ± standard deviation. All the groups were conformed to the normal distribution performing Shapiro-Wilk test. Comparison between two groups was tested by a *T* test. Three or more groups were compared by a one-way analysis of variance (ANOVA) [21]. LSD was used for a post hoc test. Statistical analysis was performed using SPSS 17.0, and *P* value (*P* < 0.05) was considered statistically significant. GraphPad Prism 6 software (GraphPad Software Inc., San Diego, USA) was used to generate graphs for our results [9].

## 3. Results

### 3.1. lncRNA STAT4-AS1 Expression Was Downregulated Accompanied by Upregulation of IL-17A Expression in Asthmatic Patients

**Figure 1:**
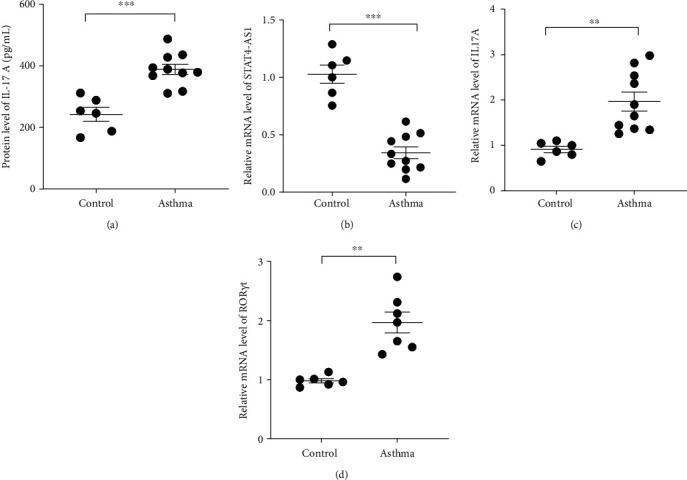
lncRNA STAT4-AS1 expression was downregulated accompanied by up-regulation of IL-17 expression in asthmatic patients. (a) The release of IL-17A in peripheral T cells of asthmatic patients was detected using ELISA. (b–d) The mRNA expression of lncRNA STAT4-AS1, IL-17A, and ROR*γ*t in peripheral blood T cells of patients with asthma was detected using Q-PCR. Three independent experiments were performed, and data are presented as the mean ± SD. ^∗∗^*p* < 0.01, and ^∗∗∗∗^*p* < 0.001.

We isolated T cell from 16 clinical samples, including 6 normal controls and 10 severe asthma patients, and then measured the expression level of STAT4-AS1 and IL-17A. Compared with the normal controls, the levels of IL-17A in supernatant were significantly higher than those in the control (Figure 1(a)). The mRNA levels of STAT4-AS1 in asthmatic patients were significantly lower than those in the normal controls (Figure 1(b)). Meanwhile, the RNA levels of IL-17A and ROR*γ*t in the asthma patients were significantly higher than those in the normal controls (Figures 1(c) and 1(d)).

### 3.2. lncRNA STAT4-AS1 Expression Was downregulated Accompanied by Upregulation of IL-17A Expression in PBMCs

**Figure 2:**
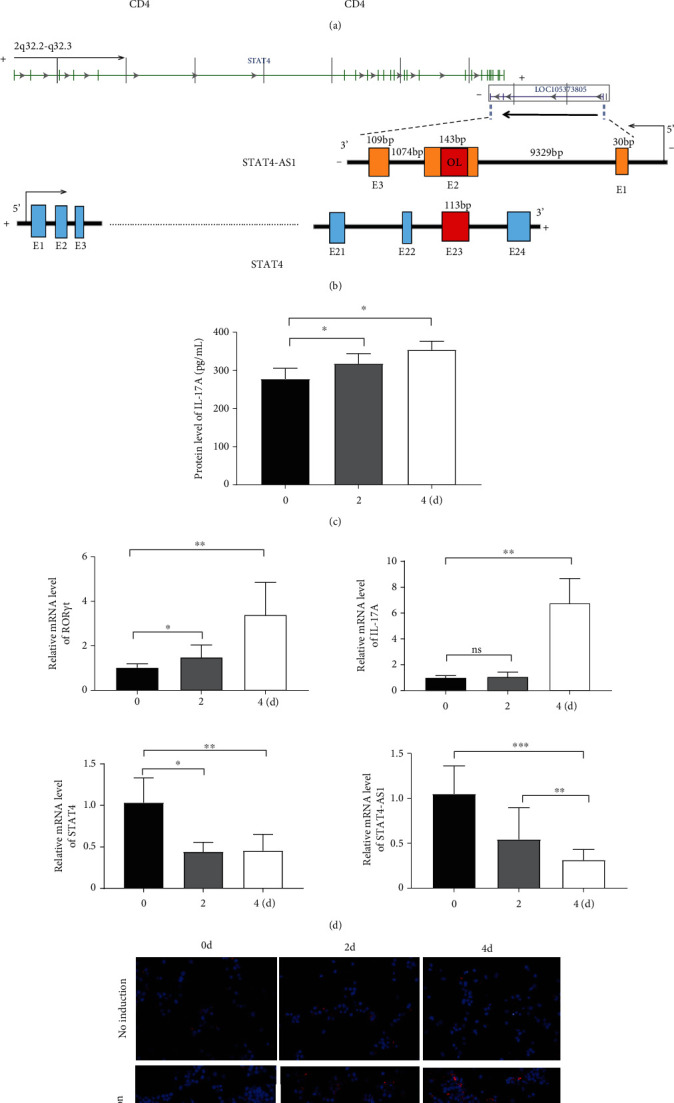
lncRNA STAT4-AS1 expression was downregulated accompanied by upregulation of IL-17 expression in PBMCs. (a) The proportion of TH17 cells on day 0 and day 3 of induced differentiation. Flow cytometry figures (left) and statistical analysis (right) of TH17 cells. (b) The upper chart of this panel shows genome organization of the stat4 gene at locus 2q32.2-q32.3. The chart shows the start and end positions of these genes as indicated on the UCSC site. Black arrows indicate transcription direction, green blocks are the exons of STAT4, and purple blocks are the exons of STAT4-AS1. The lower chart of this panel shows that the exons of STAT4 and STAT4-AS1 have an overlapping region (OL). Blocks with colors (orange, blue, and red) represent exons. Red block in STAT4 represents the 23 exon and also OL region. The genomic organization of the human STAT4-AS1 locus. “+” at both sides of strand represents the positive strand; “-” represents the negative strand. (c) The levels of IL-17A in cell supernatant were detected using ELISA. (d) The expression of IL-17A, lncRNA STAT4-AS1, STAT4, and ROR*γ*t genes was detected using qPCR, respectively. (e) The expression of intracellular ROR*γ*t protein was detected using immunofluorescence. Three independent experiments were performed, and data are presented as the mean ± SD. ^∗^*p* < 0.05, ^∗∗^*p* < 0.01, and ^∗∗∗^*p* < 0.001.

Considering the relevance of upregulated TH17 cells and their effector molecules in asthma pathogenesis, we then established a model of TH17 cells *in vitro*. PBMCs were induced into TH17 cells with TH17 polarization cytokines for four days under the activation of 2 *μ*g/mL of anti-CD3 and 5 *μ*g/mL of anti-CD28. The proportion of TH17 cells in PBMCs cells increased gradually during induction (Figure 2(a)). Based on chromosomal locations, lncRNAs are further divided into several groups: antisense, intronic, bidirectional, and intergenic, lncRNAs [39]. Through NCBI, we found that as an antisense lncRNA of STAT4, STAT-AS1 is transcribed from the STAT4 gene locus on human chromosome 2 (2q32.2-q32.3) and consists of 3 exons. Moreover, the 2^nd^ exon and the 23^rd^ exon of STAT4 have an overlapping (OL) region. As shown in Figure 2(b), red block in STAT4 gene represents the 23^rd^ exon and also OL region. The levels of IL-17A in cell supernatant gradually increased with induction time (Figure 2(c)). At the same time, the mRNA of IL-17A and ROR*γ*t also gradually increased, but STAT4-AS1 and STAT4 gradually decreased (Figure 2(d)). The result of immunofluorescence assay also showed that the level of ROR*γ*t protein gradually increased with induction time (Figure 2(e)). These results confirmed that lncRNA STAT4-AS1 was negatively correlated with IL-17A expression in PBMCs *in vitro*.

### 3.3. Downregulation of lncRNA STAT4-AS1 Promoted the Differentiation of TH17 Cells

**Figure 3:**
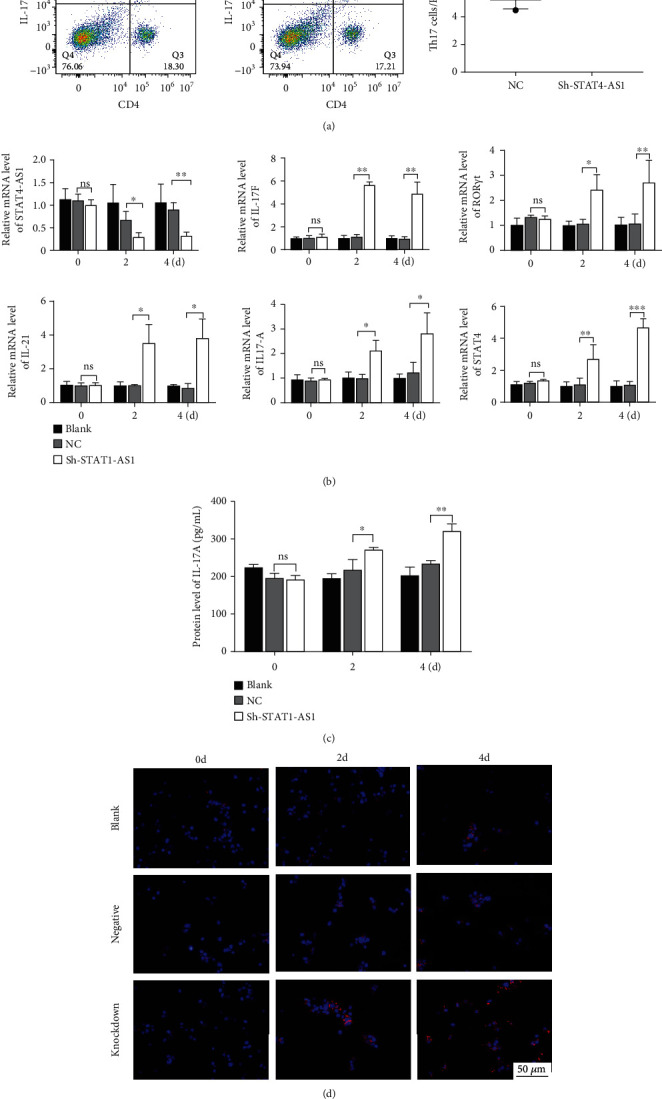
Downregulation of lncRNA STAT4-AS1 promoted the differentiation of TH17 cells. (a) The proportion of TH17 cells on day 2 and day 4 of induced differentiation. Flow cytometry figures (left) and statistical analysis (right) of TH17 cells. (b) The mRNA level of STAT4-AS1, STAT4, IL-17A, ROR*γ*t, IL-17F, and IL-21 on days 2 and 4 of induction. (c) The levels of IL-17A in cell supernatant were detected using ELISA. (d) Immunofluorescence was used to determine the protein expression of ROR*γ*t. Three independent experiments were performed, and data are presented as the mean ± SD. ^∗^*p* < 0.05, ^∗∗^*p* < 0.01, and ^∗∗∗^*p* < 0.001.

Next, lentivirus carrying sh-STAT4-AS1 was used to infect PBMCs isolated from health controls. Three days after lentivirus infection, PBMCs were induced into TH17 under the condition of TH17 differentiation *in vitro*. The result of flow cytometry showed that the proportion of TH17 cells increased gradually after induction (Figure 3(a)). On the second day of induction, the RNA level of STAT4-AS1 in the shRNA-STAT4-AS1 group decreased significantly when compared with that of the control group. However, the mRNA level of IL-17A, ROR*γ*t, IL-17F, and IL-21 increased significantly, and STAT4 showed no changes. On the fourth day of induction, all of the mRNAs mentioned above, including STAT4, were increased significantly in the sh-STAT4-AS1 group compared with those in the control group (Figure 3(b)). The levels of IL-17A in supernatant gradually increased with induction time (Figure 3(c)). Moreover, the expression of ROR*γ*t in all three groups increased gradually, while it increased significantly in the sh-STAT4-AS1 group when compared with the control group (Figure 3(d)).

### 3.4. Upregulation of lncRNA STAT4-AS1 Inhibited the Differentiation of TH17 Cells

**Figure 4:**
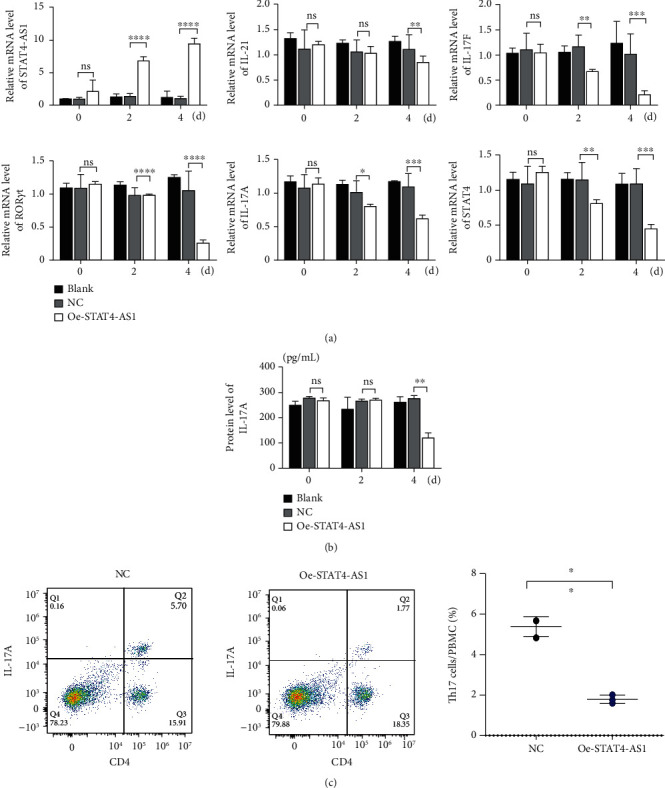
Upregulation of lncRNA STAT4-AS1 inhibited the differentiation of TH17 cells. (a) The mRNA levels of lncRNA STAT4-AS1, STAT4, IL-17A, ROR*γ*t, IL-17F, and IL-21 on day 0, day 2, and day 4 after induction. (b) The level of IL-17A in the supernatant. (c) The proportion of TH17 cells after lncRNA STAT4-AS1 overexpression. Flow cytometry figures (left) and statistical analysis (right) of TH17 cells. Three independent experiments were performed, and data are presented as the mean ± SD. ^∗^*p* < 0.05, ^∗∗^*p* < 0.01, and ^∗∗∗^*p* < 0.001.

On the other hand, we also upregulated the expression of STAT4-AS1 in PBMCs. Two days after lentivirus infection, PBMCs were induced into TH17 cells under the condition of TH17 differentiation *in vitro*. As shown in Figure 4(a), lncRNA STAT4-AS1 increased significantly with the time of infection. On the second day of induction, the mRNA level of IL-17A, ROR*γ*t, IL-17F, and STAT4 decreased in the STAT4-AS1 overexpressed group when compared with those in the NC or blank group while IL-21 showed no changes. On the fourth day of induction, all the mRNA level of IL-17A, ROR*γ*t, IL-17F, STAT4, and IL-21 decreased in the STAT4-AS1 overexpressed group (Figure 4(a)). The level of IL-17A in the supernatant of PBMCs also decreased after lncRNA STAT4-AS1 overexpression (Figure 4(b)). The result of flow cytometry showed that the proportion of TH17 cells was decreased significantly after STAT4-AS1 overexpression (Figure 4(c)).

### 3.5. lncRNA STAT4-AS1 Interacts with ROR*γ*t and Inhibits the Expression of IL-17A

**Figure 5:**
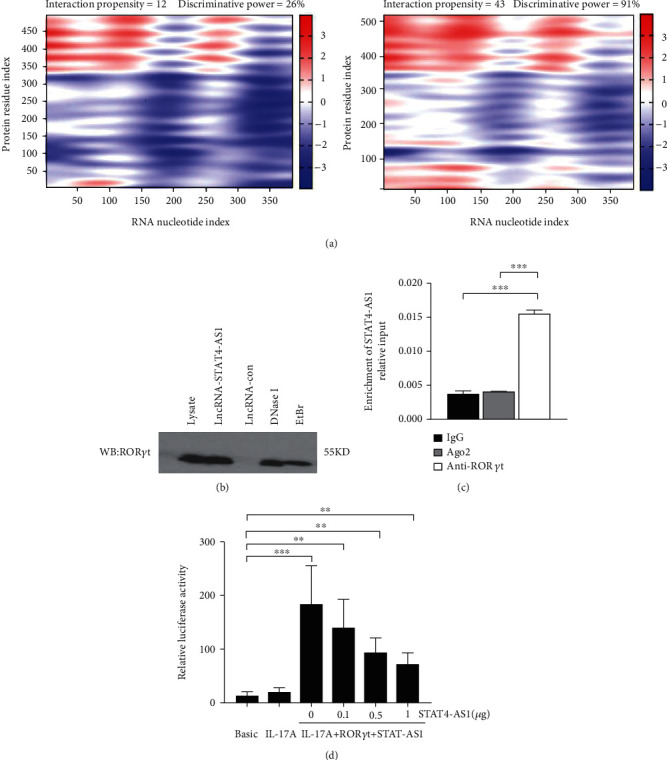
lncRNA STAT4-AS1 interacted with ROR*γ*t and inhibited IL-17 expression. (a) Bioinformatics analysis predicted results of the interaction between lncRNA STAT4-AS1 and ROR*γ*t protein. Left: the interaction between lncRNA STAT4-AS1 and ROR*γ*t protein. Right: the interaction between lncRNA STAT4-AS1 and ROR*γ*t protein isoform (ROR*γ*). (b) ROR*γ*t protein was detected in the extracts of the RNA pull-down using Western blot. lncRNA-Con was used as negative control. (c) RT-qPCR detection of lncRNA STAT4-AS1 mRNA in the immunoprecipitation by IgG, AGO2 or anti-ROR*γ*t specific antibody from PBMCs treated with TH17 polarization. IgG served as a negative control. (d) Dual luciferase reporter assay was used to confirm the inhibition of lncRNA STAT4-AS1 during ROR*γ*t-induced activation of the IL-17 promoter. Three independent experiments were performed, and data are presented as the mean ± SD. ^∗^*p* < 0.05, ^∗∗^*p* < 0.01, and ^∗∗∗^*p* < 0.001.

lncRNA STAT4-AS1 was predicted to interact with ROR*γ*t, and the interaction sites were the 50-100 nt region of lncRNA STAT4-AS1 and the 350-450 amino acid region of ROR*γ*t protein. Meanwhile, lncRNA STAT4-AS1 was also predicted to interact with ROR*γ*t isoform (ROR*γ*), and the interaction sites were the 50-150 nt and 250-300 region of lncRNA STAT4-AS1 and the 400-500 amino acid region of ROR*γ*t protein (Figure 5(a)). Using RNA pull-down with biotin-labeled lncRNA STAT4-AS1, we confirmed that lncRNA STAT4-AS1 interacted with ROR*γ*t in T cell extracts. This interaction still existed even in the presence of DNAase I or ethidium bromide (EB), suggesting that DNA was not involved in the interaction between lncRNA STAT4-AS1 and ROR*γ*t protein (Figure 5(b)). RIP assays confirmed that there was substantial enrichment of lncRNA STAT4-AS1 using anti-ROR*γ*t antibody compared to negative control. Moreover, lncRNA STAT4-AS1 was not associated with the AGO2 protein, a key component of the microRNA-containing RISC complex (Figure 5(c)). These findings strongly suggested that lncRNA STAT4-AS1 could remarkably bind with ROR*γ*t protein. It is reported that ROR*γ*t protein binds to the ROR response elements (Rore) within the Il17a promoter in mice and humans [18]. After the pGL3-IL-17, pcDNA3-ROR*γ*t, and pcDNA3-STAT4-AS1 plasmids were cotransfected into 293T cells in different combinations, we found that ROR*γ*t significantly promoted the activation of the IL-17 promoter, whereas lncRNA STAT4-AS1 inhibited the activity of the IL-17 promoter in a dose-dependent manner (Figure 5(d)).

**Figure 6:**
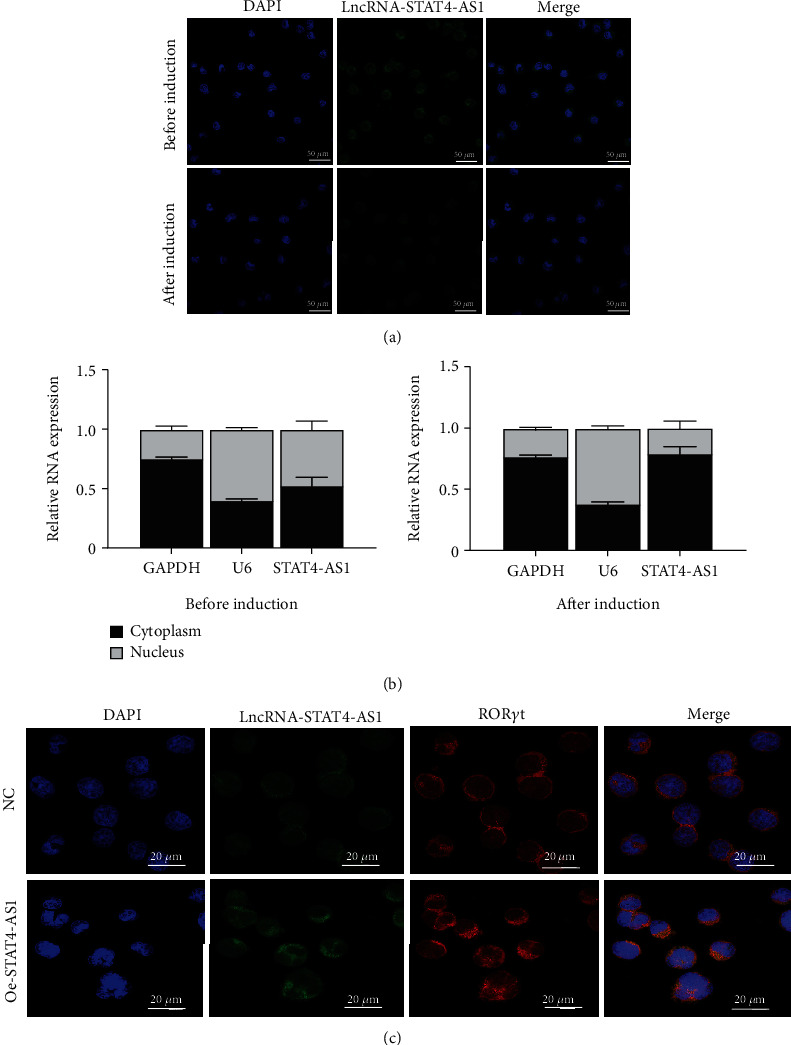
Subcellular localization of lncRNA STAT4-AS1 (*n* = 3). (a) The localization of lncRNA STAT4-AS1 in PBMCs cells before and after induction was determined by FISH. (b) Nuclear-cytoplasmic separation assay. (c) The colocalization of lncRNA STAT4-AS1 and ROR*γ*t protein in TH17 before and after overexpression of lncRNA STAT4-AS1 was determined by FISH and immunofluorescence. Three independent experiments were performed, and data are presented as the mean ± SD. ^∗^*p* < 0.05, ^∗∗^*p* < 0.01, and ^∗∗∗^*p* < 0.001.

To investigate how lncRNA STAT4-AS1 inhibit IL-17 promoter activity, we used FISH and nuclear-cytoplasmic separation assay to analyze the subcellular localization of lncRNA STAT4-AS1 in PBMCs before and after induction towards TH17. The results indicated that before induction, lncRNA STAT4-AS1 was present in both the cytoplasm and nucleus; however, after induction, the expression of STAT4-AS1 decreased significantly, and its proportion in the cytoplasm was significantly increased (Figures 6(a) and 6(b)). The colocalization of lncRNA STAT4-AS1 and ROR*γ*t was also examined in PBMCs before and after overexpression of lncRNA STAT4-AS1. The results showed that after overexpression of STAT4-AS1, the lncRNA STAT4-AS1 was significantly increased in the cytoplasm when compared to that of nucleus. ROR*γ*t also collected in the cytoplasm indicating that lncRNA STAT4-AS1 bound to ROR*γ*t protein primarily in the cytoplasm (Figure 6(c)). Therefore, we concluded that lncRNA STAT4-AS1 binding with ROR*γ*t protein decreased the enrichment of ROR*γ*t protein at the promoter region of IL-17A, thus inhibiting the transcription of IL-17A.

## 4. Discussion

As an important subset of CD4^+^ T lymphocytes, TH17 cells protected the mucosal surface mainly by secreting IL-17 and play a pivotal role in host defense against pathogens such as fungi and extracellular bacteria [34, 35]. However, TH17 differentiation disorder produced excess IL-17A that could induce autoimmune tissue damages and inflammatory diseases [15, 29]. Asthma is a disease characterized by uncontrolled airway inflammatory response; therefore, immune imbalance is the central link in the pathophysiological mechanism of asthma, and TH17 cells play an important role in the development of asthma.

IL-17A and IL-23 are key cytokines for TH17 cell development and activation. In clinical trials, the antibodies of IL-23p19, IL-23p40, IL-17A, and IL-17RA have shown promising efficacy in the treatment of many autoimmune diseases including psoriasis, ankylosing spondylitis, and multiple sclerosis [26, 46]. However, blocking IL-17A or IL-17RA is ineffective or even harmful in the treatment of Crohn's disease [12]. This may be due to the blocking of a specific cytokine that may inhibit TH17-mediated inflammation which is insufficient and several other cytokines produced by TH17 cells also play a key role in inflammation. However, the combined blocking of multiple cytokines will cause high risk to normal cells in the body. Recently, the antagonists of TH17 transcriptional regulator have been proposed as new potential therapies for TH17-mediated diseases.

TH17 cell differentiation is regulated by an important set of transcription factors, including ROR*γ*t, STAT3, IRF4, and BATF [46]. Among these key transcription factors, ROR*γ*t is only highly expressed in TH17 cells [14], so it may be an ideal therapeutic target. Several molecules targeting ROR*γ*t have been discovered, and they have shown efficacy in EAE, experimental colitis, experimental arthritis, and psoriasis-like skin inflammation models [36, 43, 44]. These studies suggest that ROR*γ*t is clinically relevant as a therapeutic target for TH17-related diseases and is even a better therapeutic option than targeting cytokines or receptors.

Many studies have demonstrated that lncRNA function can serve as critical regulators of cell type-specific effector programs, often in concert with critical lineage-specifying transcription factors [6]. In Th1 cells, the lncRNA linc-MAF-4 represses expression of the Th2 cell transcription factor MAF to promote T cell differentiation toward the Th1 cell lineage. lnc-MAF-4 recruits the chromatin remodelers EZH2-and LSD1 to place repressive chromatin marks on the Th2 cell transcription factor MAF promoter and repress its transcription, which promoted T cell differentiation toward the Th1 cell lineage [6, 33]. lncRNAs can also regulate the development and activation of TH17 cells by regulating the expression of ROR*γ*t protein. lncRNA XLOC_000261 negatively regulates the ROR*γ*t protein of TH17 cells in Crohn's disease [4].

**Figure 7:**
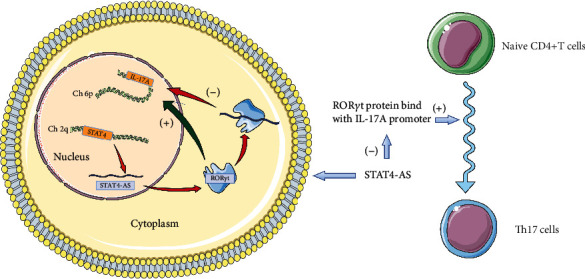
The mechanism of lncRNA STAT4-AS1 on TH17 differentiation.

In our study, lncRNA STAT4-AS1 has proven evidence to control gene expression through direct interaction with ROR*γ*t protein and subsequent inhibition of the activation of IL-17 promoter (Figure 7). Moreover, lncRNA STAT4-AS1 mainly colocalized with ROR*γ*t protein in the cytoplasm and affect its translocation into the nucleus. Two mechanisms have been reported for cytoplasmic lncRNA action: (i) sequestration of microRNA to restore mRNA translation as a competing endogenous RNA or (ii) controlling the posttranslational modification of transcription factors [40]. However, RIP revealed that lncRNA STAT4-AS1 was not associated with the Ago2 protein, a key component of the microRNA-containing RISC complex [11, 38]. Therefore, we focus on the second critical mechanisms. ROR*γ*t, as the master transcription factor of TH17 cells, is modified by multiple posttranslational mechanisms, including ubiquitination, acetylation, SUMOylation, and phosphorylation [18]. The next step is to investigate whether lncRNA STAT4-AS1 affects the posttranslational modification of ROR*γ*t and to find out the specific binding sites.

In addition, lncRNA STAT4-AS1 was derived from STAT4 gene. Antisense lncRNA is often associated with the expression of positive-sense chain genes. The RNA level of STAT4 was increased when STAT4-AS1 was knocked down, and the RNA level of STAT4 was decreased when STAT4-AS1 was overexpressed. This indicates that STAT4-AS1 may be originated from STAT4 but give a feedback suppression to the expression of STAT4.

Overall, lncRNA STAT4-AS1 combined with ROR*γ*t protein could inhibit the activation of IL-17 promoter and ultimately inhibits TH17-related gene expression and TH17 cell differentiation. The expression mechanism of lncRNA STAT4-AS1, its binding site to ROR*γ*t, its effect on the RNA level of STAT4, and whether the regulation of TH17 by lncRNA STAT4-AS1 is specific will be the focus of our next study. Consequently, this study demonstrates the potential of lncRNA STAT4-AS1 as a target for clinical diagnosis, prognosis, phenotype, and treatment of TH17-mediated diseases such as refractory asthma.

## Data Availability

The data used and/or analyzed during the current study are available from the corresponding authors on reasonable request.

## Supplementary Materials

Supplementary Materials Supplemental Table 1: the clinical information of all participants. Supplemental Table 2: primers used for RT-qPCR.
