# Supplementary material for: lncRNA STAT4-AS1 Inhibited TH17 Cell Differentiation by Targeting RORγt Protein
Source: J Immunol Res. 2022 Apr 28;2022:8307280. doi: 10.1155/2022/8307280 (PMC9071868; doi:10.1155/2022/8307280)
Supplement: Supplementary Materials — Supplemental Table 1: the clinical information of all participants. Supplemental Table 2: primers used for RT-qPCR. [file 8307280.f1.zip › 8307280.f1.doc]

**Supplemental Table 1. Characteristics of all subjects**

|  | Healthy subjects (n = 6) | Asthma (n = 10) |
| --- | --- | --- |
| Age (y) | 55.8 ±3.2 | 56.1 ±5.5 |
| BMI(kg/m^2^) | 23.04±3.87 | 22.89±4.19 |
| FEV1 (L) | 3.32 ± 0.19 | 1.45 ± 0.21** |
| FEV1 (%) predicted | 96.11 ±12.17 | 55.38 ±13.55** |
| FVC (L) | 4.03 ± 0.41 | 2.85 ± 0.22** |
| FEV1/FVC (%) | 81.70±5.94 | 49.76±10.17** |
