# Supplementary material for: lncRNA STAT4-AS1 Inhibited TH17 Cell Differentiation by Targeting RORγt Protein
Source: J Immunol Res. 2022 Apr 28;2022:8307280. doi: 10.1155/2022/8307280 (PMC9071868; doi:10.1155/2022/8307280)
Supplement: Supplementary Materials — Supplemental Table 1: the clinical information of all participants. Supplemental Table 2: primers used for RT-qPCR. [file 8307280.f1.zip › 8307280.f2.doc]

| Primers | Sequences | |
| --- | --- | --- |
| RORγt | Forward | 5'-GAGGAAGTGACTGGCTACCAGA-3' |
|  | Reverse | 5'-GCACAATCTGGTCATTCTGGCAG-3' |
| IL17A | Forward | 5'-CGGACTGTGATGGTCAACCTGA-3' |
|  | Reverse | 5'-GCACTTTGCCTCCCAGATCACA-3' |
| IL-17F | Forward | 5'-AACCAGCGCGTTTCCATGTCAC-3' |
|  | Reverse | 5'-GAGCATTGATGCAGCCCAAGTTC-3' |
| IL-21 | Forward | 5'-CCAAGGTCAAGATCGCCACATG-3' |
|  | Reverse | 5'-TGGAGCTGGCAGAAATTCAGGG-3' |
| STAT4 | Forward | 5'-CAGTGAAAGCCATCTCGGAGGA-3' |
|  | Reverse | 5'-TGTAGTCTCGCAGGATGTCAGC-3' |
| LncRNA-STAT4-AS1 | Forward | 5'-AACACCGCATACACACTTGGAGAC-3' |
|  | Reverse | 5'-CAGACTTGCAGCCACCTGACTTC-3' |
| GAPDH | Forward | 5'-CAGGAGGCATTGCTGATGAT-3' |
|  | Reverse | 5'-GAAGGCTGGGGCTCATTT-3' |

**Supplemental Table 2. The used primer sequences in RT-qPCR**
